# Systematic review regarding metabolic profiling for improved pathophysiological understanding of disease and outcome prediction in respiratory infections 

**DOI:** 10.1186/s12931-015-0283-6

**Published:** 2015-10-15

**Authors:** Manuela Nickler, Manuel Ottiger, Christian Steuer, Andreas Huber, Janet Byron Anderson, Beat Müller, Philipp Schuetz

**Affiliations:** Medical University Department, Division of General Internal and Emergency Medicine, Kantonsspital Aarau, Aarau, Switzerland; Department of Laboratory Medicine, Kantonsspital Aarau, Aarau, Switzerland; Principal, Medical Linguistics Consulting, North Olmsted, OH USA; University Department of Medicine, Kantonsspital Aarau, Tellstrasse, CH-5001 Aarau, Switzerland

**Keywords:** Lower respiratory tract infection, Pneumonia, Chronic obstructive pulmonary disease, Mortality prediction, Steroid hormones, Biogenic amines, Trimethylamine-*N*-oxide, Intermediates energy metabolism, Monosaccharides, Oxidative status, Glycerophospholipids, Sphingolipids, Acylcarnitines

## Abstract

Metabolic profiling through targeted quantification of a predefined subset of metabolites, performed by mass spectrometric analytical techniques, allows detailed investigation of biological pathways and thus may provide information about the interaction of different organic systems, ultimately improving understanding of disease risk and prognosis in a variety of diseases. Early risk assessment, in turn, may improve patient management in regard to cite-of-care decisions and treatment modalities. Within this review, we focus on the potential of metabolic profiling to improve our pathophysiological understanding of disease and management of patients. We focus thereby on lower respiratory tract infections (LRTI) including community-acquired pneumonia (CAP) and chronic obstructive pulmonary disease (COPD), an important disease responsible for high mortality, morbidity and costs worldwide. Observational data from numerous clinical and experimental studies have provided convincing data linking metabolic blood biomarkers such as lactate, glucose or cortisol to patient outcomes. Also, identified through metabolomic studies, novel innovative metabolic markers such as steroid hormones, biogenic amines, members of the oxidative status, sphingo- and glycerophospholipids, and trimethylamine-*N*-oxide (TMAO) have shown promising results. Since many uncertainties remain in predicting mortality in these patients, further prospective and retrospective observational studies are needed to uncover metabolic pathways responsible for mortality associated with LRTI. Improved understanding of outcome-specific metabolite signatures in LRTIs may optimize patient management strategies, provide potential new targets for future individual therapy, and thereby improve patients’ chances for survival.

## Introduction

### Metabolomics

The metabolome can be considered the “quantitative complement of all the low molecular weight molecules (<1500 amu) in a particular physiological or developmental state“ of a cell, tissue or organism [1]. Metabolomics is defined as “*the analysis of the whole metabolome under a given set of physiological, environmental and/or clinical conditions*“ [2]. Metabolite profiling through targeted quantification of a predefined subset of metabolites of the metabolome [3] allows analysis of metabolic pathways, thereby clarifying the interaction of different organic systems. While proteomics is the study of proteins made by the organism and the conditions under which the organism makes them, metabolomics is a more systematic study of the biochemical fingerprints that result of cellular processes.

Mass spectrometry (MS) is a sensitive tool that characterizes and quantifies metabolites in a biological sample [4]. Since metabolomics requires proper separation of the different compounds to be analyzed, chemical separation techniques such as gas chromatography (GC) and liquid chromatography (LC) or capillary electrophoresis (CE) can be combined with MS detection. Analytical techniques such as LC-MS, GC-MS, CE-MS, and matrix-assisted laser desorption ionization-MS (MALDI-MS) may therefore expand coverage of the metabolome [5]. MS is now a routine diagnostic instrument in clinical laboratories [6].

Biological information derived from these techniques can provide accurate and clinically useful diagnostic capability for the management of diseases [7, 8] by mapping disease risk to metabolic pathways [9]. Previously, many studies have focused on risk prediction in patients with lower respiratory tract infections (LRTI) including community-acquired pneumonia (CAP) and case (COPD) because such information may have direct consequences in patient management [10–13]. Early risk prediction may improve site-of-care decisions (i.e., ICU treatment vs. hospital ward vs. outpatient) as well as treatment modalities (i.e., antibiotic drugs) [11, 14, 15]. Indeed, improved understanding of metabolic interactions in LRTI patients may enhance prediction of outcomes and provide targets for individual therapy, which in turn could facilitate personalized patient management (Fig. 2) [16–18].

### Prediction of mortality in community-acquired pneumonia

CAP is the leading cause of infectious death [19], and of high mortality, morbidity, and costs worldwide [20–22]. Patients with CAP manifest high short-term mortality [23, 24] but also substantial long-term morbiditiy [25–28]. To predict all-cause mortality within 30 days, the pneumonia severity index (PSI) is a validated tool that categorizes CAP patients within distinct risk classes (I-V) [29]. In Europe, the CURB-65 score (Confusion, Urea, Respiratory rate, Blood pressure, Age >65 years) is used for the same purpose [30]. Several clinical studies show that measuring specific inflammatory or metabolic blood biomarkers, including among others proadrenomedullin (proADM) [14, 15, 31–33] and procalcitonin (PCT) [26, 34–36], provides prognostic information and possibly improves short-term risk stratification and management decisions in CAP compared to the use of clinical risk scores alone [37]. Pneumonia not only increases short-term mortality; clinical trials also found greater long-term mortality rates in patients who survived an initial CAP episode, compared to the general population and to patients who have other infections [28, 38, 39]. These observations suggest that CAP is a poor prognostic indicator of long-term outcomes. Particularly, it has been hypothesized that cardiovascular events that are triggered by an episode of respiratory infection may be responsible for the excess mortality observed in large long-term CAP cohorts. For this reason, cardiovascular biomarkers (such as natriuretic peptides) may also help in the identification of high risk LRTI patients who need close monitoring and follow-up [28]. The metabolomics approach for LRTI thus may also look into metabolites that have been linked to cardiovascular disease as these conditions are being linked very closely.

### Prediction of mortality in patients hospitalized with exacerbated chronic obstructive pulmonary disease

Prediction of outcomes in chronic obstructive pulmonary disease (COPD) is challenging. Three published observational studies suggest that the inflammatory blood biomarker ProADM independently predicts medium-term all-cause mortality in patients with stable [40, 41] or exacerbated COPD. Additionally, measurement of ProADM combined with the clinical parameters of body mass index, airflow obstruction, dyspnea and exercise capacity index (BODE) [42] improves the predictive power of medium-term all-cause mortality compared to the use of BODE alone [40]. A recent observational clinical cohort study demonstrated that ProADM levels are independent predictors of 5- to 7-year all-cause mortality, and may enhance long-term prognostic accuracy of demographic and clinical variables in patients hospitalized for exacerbation of pneumonic or non-pneumonic COPD [32]. Similar to CAP, in COPD cardiovascular disease may be responsible for large portions of the high mortality associated with this disease. Again, the metabolomic approach in COPD may therefore also consider interesting metabolites associated with adverse cardiovascular outcomes.

## Aims of the review and methodology

The goal of the present review is to investigate wether new metabolic blood biomarkers help optimizing identification of patients at risk for poor outcome in CAP and exacerbated COPD. We discuss nine metabolite classes: steroid hormones, biogenic amines, intermediate energy metabolism, monosaccharides, trimethylamines, oxidative status, sphingolipids, glycerophospholipids, and acylcarnitines. Each section will begin with a brief description of pathways and known physiological effects, followed by a summary of observational data regarding the analyte’s role in predicting all-cause mortality. We focus on patients with LRTI which is responsible for high mortality, morbidity and costs worldwide. Yet, if no data are available in strictly LRTI patients, we also discuss these markers in other diseases namely cardiovascular disease which may account for a large proportion of deaths attributable to LRTI.

**Fig. 1:**
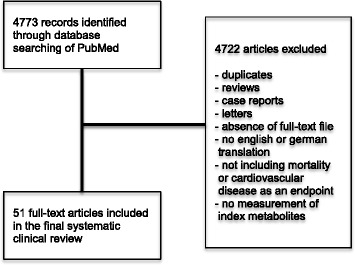
Study flow diagram

Literature discussed in this review was in part identified through a systematic literature search of English-language publications indexed in PubMed in March 2015 under the terms “lower respiratory tract infection” or “pneumonia” or “chronic obstructive pulmonary disease” combined with “mortality” with or without “prediction” and/or “cardiovascular risk” together with any of the terms “steroid hormones” or “biogenic amines” or “trimethylamine-N-oxide” or “intermediates of energy metabolism” or “oxidative status” or “monosaccharides” or “sphingolipids” or “glycerophospholipids” or “acylcarnitines”. Additionally, more specific keywords were used: “aldosterone”, “androstenedione”, “androsterone”, “corticosterone”, “cortisol”, “cortisone”, “dehydroepiandrosterone”, “11-deoxycorticosterone”, “dihydrotestosterone”, “11-deoxycortisol”, “dehydro-epiandrosterone-sulfate”, “betaestradiol”, “estrone”, “eiocholanolone”, “17-alpha-hydroxyprogesterone”, “progesterone”, “testosterone”, “acetylornithine”, “asymmetric dimethylarginine”, “symmetric dimethylarginine”, “alpha-aminoadipic acid”, “carnosine”, “creatinine”, “histamine”, “kynurenine”, “methioninesulfoxide”, “nitrotyrosine”, “cis-4-hydroxyproline”, “trans-4-hydroxyproline”, “phenylethylamine”, “putrescine”, “sarcosine”, “serotonin”, “spermidine”, “spermine”, “taurine”, “dopamine”, “DOPA”, “lactate”, “3-phosphoglycerate”, “alpha-ketoglutaric acid”, “adenosine-3′,5′-cyclic monophosphate”, “arginine”, “aspartate”, “dihydroxyacetonephosphate”, “fumarate”, “glutamate”, “hexose”, “hexosephosphate”, “pentosephosphate”, “pyruvate”, “succinate”, “tetrosephosphate”, “hexoses”, “glutathione”, “glutathione-disulfide”, “cysteine”, “cystine”, “homocysteine”, and “homocystine”. Additional systematic reviews of the topic were included if they provided new insights and evidence. As shown in Fig. 1, 4722 of  4773 articles were excluded due to duplication, absence of full text or inappropriate contents. Letters and case reports were also excluded.

**Fig. 2:**
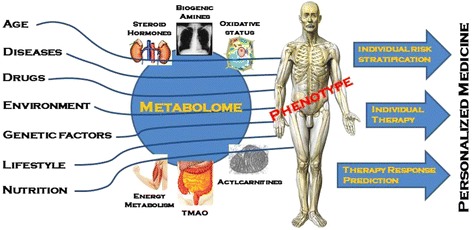
Metabolomics to improve outcome prediction in LRTI. Figure 2 shows the influence of different factors such as age, diseases, drugs, environment, genetic factors, lifestyle and nutrition on endogenous metabolome which defines the human phenotype. The knowledge of metabolic interactions may provide individual risk stratification, prediction of therapy response as well as new targets for future individual therapy and therefore a form of personalized medicine. (Abbr.: TMAO, trimethylamine-*N*-oxide)

## Promising metabolic biomarkers for prediction of all-cause mortality

### Steroid hormones

#### Pathway and physiological role

Only few tissues contribute substantially to *de novo* steroid biosynthesis: primarily the adrenal glands, the gonads, and the placenta [43]. All steroid hormones are derived from cholesterol. Cholesterol enters the cell via lipoprotein-binding and is stored in cytoplasmic vacuoles. After binding of adrenocorticotropic hormone (ACTH) to its receptor (melanocortin receptor type 2, MC2R) on adrenal cells, cholesterol is mobilized and thus available for steroid synthesis. Follicle-stimulating hormone (FSH) is critical in estradiol synthesis before and during ovulation and Luteinizing hormone (LH) for synthesis of progesterone. In the placenta, steroid biosynthesis is dependent on human chorionic gonadotropin (hCG) in early gestation [43].

One of the first measurable physiopathological reactions to infection is the activation of the hypothalamo-pituitary-adrenal (HPA) axis via stimulation of the central noradrenergic stress system by cytokines and other mediators that are released in response to inflammation [44–46]. Under healthy conditions, cortisol secretion is synchronized with dehydroepiandrosterone (DHEA) in response to the release of ACTH and corticotropin-releasing hormone (CRH), whereas in critically ill patients the circadian pattern of cortisol is lost [47] and DHEA and dehydroepiandrosterone-sulfate (DHEA-S) decrease [48]. Although the physiological role of DHEA and DHEA-S is not completely understood, there are clear indications that they modulate the immune response and influence proinflammatory and anti-inflammatory cytokine release [48–52]. In severely ill pneumonia patients, an intra-adrenal shift from DHEA-S to cortisol production occurs, which might be a potentially life-saving process during critical illness and thus important for survival in CAP [53]. Estradiol induces activation of macrophages and production of proinflammatory cytokines and chemokines from inflammatory cells [54–56], and testosterone has suppressive effects on immune responses, thereby inducing increased suceptibility to infection [57]. In autoimmune diseases progesterone shows proinflammatory and anti-inflammatory effects [58].

#### Current research (Table 1)

Table 1 lists several studies reporting that increased cortisol (free and total) levels measured at initial hospital presentation are independent predictors of in-hospital and short-term all-cause mortality in hospitalized patients with mild to severe CAP [53, 59–62]. Interestingly, the predictive accuracy of free cortisol (FC) is equal to that of total cortisol (TC) in pneumonia, independent of serum albumin levels [59]. The prognostic accuracy of cortisol in predicting mortality is equal to that of the PSI, but higher compared to routinely measured laboratory parameters such as CRP, PCT or leukocytes, which do not show significant differences (p > 0.05) [59–61]. Kolditz et al. observed that serum cortisol levels may noticeably improve the predictive power of the CURB-65 score alone as regards short-term mortality [60, 61]. Salluh et al. demonstrated that compared to baseline serum cortisol levels, which are proven independent mortality predictors, neither postcorticotropin cortisol nor Δ-cortisol has notable predictive power in severe CAP [62]. A recent animal study of canines suffering from staphylococcus aureus pneumonia showed that ACTH stimulation induces a pronounced correlation between all-cause mortality and cortisol (free and total) levels and Δ-cortisol [63].

**Table 1:** Summary of selected literature relevant to steroid hormones in all-cause mortality prediction

First author, year, reference	Marker	Study type	Study population	Key findings	Limitations
Mueller et al., 2014, [53]	- Cortisol	Single-center, prospective observational cohort study (6-week follow-up)/secondary chart analysis of a single-center, randomized controlled interventional study	179 prospectively recruited patients hospitalized with CAP	- In age and gender adjusted logistic regression analysis, cortisol (OR: 2.8; 95 % CI: 1.48–5.28, *p* = 0.002) and DHEA (OR: 2.62; 95 % CI: 1.28–5.34, *p* = 0.008), but not DHEA-S and none of the different ratios (cortisol/DHEA, cortisol/DHEA-s, DHEA/DHEA-S) were associated with increased 6-week all-cause mortality; the discriminatory accuracy in ROC analysis (AUC) to predict mortality was 0.74 (95 % CI: 0.60–0.88) for cortisol, 0.61 (95 % CI: 0.46–0.75) for DHEA and 0.72 (95 % CI: 0.62–0.81) for the PSI	- Single-center study
	- DHEA				- Secondary chart analysis of remaining blood samples; the study had not been prospectively designed to use adrenal hormone concentrations as a primary endpoint [139]
	- DHEA-S				- Cortisol, DHEA-S and DHEA levels were measured at different times during the day
Christ-Crain et al., 2007, [59]	- Free cortisol	Single-center, prospective observational cohort study (6.9 +/− 1.9-week follow-up)/secondary chart analysis of a single-center, randomized controlled interventional study	278 prospectively recruited patients presenting to the emergency department with CAP	- Initial TC and FC levels were significantly higher in non-survivors than in survivors (*p* < 0.001 for TC and *p* = 0.004 for FC); the area under the receiver operating characteristic curve (AUC) to predict death was 0.76 (95 % CI, 0.70–0.81) for TC and 0.69 (95 % CI, 0.63–0.74) for FC, whereas the AUC of the PSI was 0.76 (95 % CI, 0.70–0.81) to predict mortality	- This study had not been prospectively designed to use cortisol concentrations as a primary endpoint, and only single initial cortisol levels were measured [139]
	- Total cortisol			- The prognostic accuracy of FC was not higher than the one for TC (*p* = 0.12)	- Cortisol levels were measured at different timepoints during the day
					- No assessment of adrenal function
				- TC and FC levels were independent mortality predictors in pneumonia; the prognostic accuracy of total cortisol equaled the predictive power of the PSI	
Kolditz et al., 2012, [60]	Cortisol	Multicenter, prospective observational cohort study (patients recruited from CAPNETZ) (30-day follow-up)	984 hospitalized CAP patients, recruited from a multicenter national CAP-study in Germany	- After a follow-up period of 30 days initial serum cortisol levels were significantly higher in non-survivors than in survivors (*p* < 0.001); the AUC to predict 30-day mortality was 0.70 (cut-off serum cortisol value 795 nmol/l)	- No correction for concomitant steroid medication, due to absent data
				- Predictive accuracy of the CURB-65 score alone (AUC 0.76) was significantly improved by combining with serum cortisol levels (AUC 0.81, *p* = 0.001)	- Since blood samples were taken at time of first contact, cortisol levels were measured at different time points during the day; however, during infectious diseases and thus increased stress levels the circadian pattern of cortisol production is often lost [140]
				- Survival analysis by Kaplan-Meyer curves demonstrated a significantly different survival within cortisol-quartiles (*p* < 0.001), which persisted within individual CURB-65 classes (*p* = 0.002–0.003).	
					- No assessment of adrenal function
Kolditz et al., 2010, [61]	- Cortisol	Single-center, prospective observational cohort study (30-day follow-up)	59 adult patients hospitalized with CAP	- After a follow-up period of 30 days cortisol was significantly higher in non-survivors compared to survivors (*p* = 0.009) and was an independent predictor of short-term mortality (OR 1.002, 95 % CI 1.000–1.004, *p* = 0.04)	- Small sample size
	- DHEA			- The prognostic accuracy of serum cortisol (AUC 0.83 (0.62–1.0); cut-off serum cortisol: 734 nmol/l) was similar to that of the PSI (AUC 0.86 [0.75–0.97]) and superior to that of the CURB-65 score (AUC 0.68, 95 % CI 0.50–0.85) in predicting 30-day mortality	- Single-center study
	- DHEA-S				- No measurements of serum levels of free cortisol
					- Cortisol, DHEA and DHEA-S levels were measured at different timepoints during the day, which could limit the prognostic accuracy of one single cortisol value
				- Serum cortisol levels were independent 30-day mortality predictors in pneumonia, whereas DHEA and DHEA-S showed no significant difference between survivors and non-survivors (*p* = 0.16 and *p* = 0.70)	
					- DHEA-S shows age-dependent differences in secretion levels
Salluh et al., 2008, [62]	Total cortisol	Single-center, prospective observational cohort study (follow-up until death (in-hospital mortality) or hospital discharge)	72 patients with severe CAP admitted to the ICU	- TC levels were significantly higher in non-survivors compared to survivors (*p* = 0.003 for TC), whereas postcorticotropin cortisol levels and Δ-cortisol achieved no significant difference between survivors and nonsurvivors (p ≥ 0.05, exact value not shown)	- Small sample size with a mortality rate of only 16.7 % (12 patients), leading to low statistical power
				- Increases in mortality rates were observed across all quartiles of baseline TC levels	- Single-center study
				- The AUC to predict mortality was 0.77 (95 % CI, 0.64–0.90; *p* = 0.002; cut-off 25.7 μg/dl) for TC, 0.60 for delta-cortisol (95 % CI 0.42–0.78; *p* = 0.24), 0.58 for postcorticotropin cortisol (95 % CI 0.43–0.74; *p* = 0.33), 0.71 for CURB-65 (95 % CI 0.57–0.86; *p* = 0.01) and 0.71 for APACHE II score (95 % CI 0.56–0.86; *p* = 0.01), whereas baseline TC achieved a significantly higher AUC than postcorticotropin cortisol (*p* = 0.03)	- No data about patients with severe immunosuppression
					- No measurement of free cortisol levels
				- Kaplan-Meier curves demonstrated serum baseline cortisol (TC) concentration of >25.7 μg/dl on presentation to have a significantly higher risk of death (log rank test, *p* < 0.001) compared to baseline cortisol levels below this cut-off (*p* = 0.019)	- Measurement of cortisol levels at different timepoints during the day, potentially limiting the prognostic accuracy of one single cortisol value
					- Potential confounding due to treatment with corticosteroids in the study population
				- In univariate analysis, baseline cortisol, CURB-65 and APACHE II score were predictors of short-term mortality	
Cortés-Puch et al., 2014, [63]	- Total cortisol	Experimental, prospective animal study (96-hour follow-up)	101 canine suffering from S. aureus pneumonia-induced sepsis	- At 10 h after onset of sepsis total and free cortisol levels and change in cortisol levels after exogenous ACTH administration (delta cortisol) showed no significant difference between survivors and non-survivors (*p* = 0.75 for TC, *p* = 0.80 for FC, *p* = 0.64 for delta cortisol after ACTH stimulation), whereas ACTH levels correlated significantly, but weakly, with mortality 10 h after onset of sepsis (*p* = 0.04)	- Low sample size
	- Free cortisol				- Cortisol responses may partly be attributed to sedation, intubation, mechanical ventilation, and repeated ACTH stimulation testing
				- At 24 h total and free cortisol, ACTH levels as well as delta cortisol after ACTH stimulation correlated significantly with	
	- ACTH				
	- Aldosterone				
Ohlsson et al., 2010, [65]	- DHEA	Prospective observational population-based cohort study (mean 4.5-year follow-up)	2644 Swedish men from the Swedish multicenter Osteoporotic Fractures in Men cohort	- Low levels of DHEA-S and DHEA (quartile 1 vs. quartiles 2–4) predicted all-cause mortality (multivariate adjusted HR 1.54, 95 % CI 1.21–1.96 for DHEA-S; HR 1.48, 95 % CI 1.17–1.88 for DHEA)	- Single measurement of DHEA and DHEA-S at different timepoints during the day with subsequent possible diurnal variation in serum DHEA and DHEA-S levels
	- DHEA-S				
				- Age-adjusted risk of all-cause mortality was increased in men within Q1 of DHEA and DHEA-S levels compared with men within the individual quartiles 2, 3 and 4 (Q2, Q3, Q4) (Q1: HR 1.00 (referent); DHEA: Q2 vs. Q1 HR 0.69, Q3 vs. Q1 HR 0.66, Q4 vs. Q1 HR 0.60; DHEA-S: Q2 vs. Q1 HR 0.69, Q3 vs. Q1 0.71, Q4 vs. Q1 0.60)	- No data about treatment with corticosteroids or other hormones, which might alter mortality risk and/or DHEA and DHEA-S levels
Hsu et al., 2012, [66]	DHEA-S	Single-center, prospective observational cohort study (mean follow-up time: 38.2 +/− 20.4 months)	200 CKD patients (men) on hemodialysis (HD) more than 6 months	- Low plasma DHEA-S levels (cut-off 790 ng/ml) showed significant association with increased all-cause mortality (HR 3.667, 95 % CI 1.710–7.909; *p* = 0.001) in hemodialysis men, but not in women (data not shown)	- Single-center study
					- No assessmet of testosterone levels as a precursor of DHEA-S
				- Multivariate Cox regression analysis adjusted for age, comorbidities such as diabetes mellitus, chronic heart failure, COPD, CRP as well as for albumin and creatinine demonstrated an independent association between low plasma DHEA-S levels and all-cause mortality in HD men (HR 2.93, 95 % CI 1.09–7.89; *p* = 0.033)	- Plasma DHEA-S levels were analyzed from one single pre-dialysis sample
Feng et al., 2014, [67]	- Estradiol	Single-center, prospective observational cohort study (28-day follow-up)	107 clinically diagnosed pneumonia-related septic shock patients	- Serum levels of progesterone and estradiol were significantly higher in non-survivors compared to survivors (*p* < 0.001), whereas testosterone levels were similar in both groups (*p* = 0.74)	- Single-center study
	- Progesterone				- Most of the patients participating in the study were male
	- Testosterone			- The discriminatory accuracy in ROC analysis (AUC) to predict 28-day mortality was 0.87 (*p* < 0.001) for the APACHE II score (cut-off 26 points), 0.705 (*p* < 0.001) for estradiol (cut-off 40 pg/ml), 0.713 (*p* < 0.001) for progesterone (cut-off 1.03 ng/ml) and 0.518 (*p* = 0.74) for testosterone (cut-off 4.4 ng/ml)	- No enrollment of premenopausal women
					- Blood sample collection for hormone measurements only on the first day of septic shock (no investigation of the dynamic of serum sex hormone levels during the course of disease)
Shores et al., 2014, [68]	- Total testosterone	Prospective observational cohort study (9-year follow-up)	1032 men in the Cardiovascular Health Study (CHS)	- Adjusted for age and cardiovascular risk factors, low DHT (<25 ng/dl) and cFDHT (<0.13 ng/dl) levels showed significant associations with higher all-cause mortality, even if adjusted for cardiovascular risk factors (HR 1.31, 95 % CI 1.04–1.65 for DHT; HR 1.72, 95 % CI 1.25–2.37 for calculated free DHT; *p* < 0.001 for both)	- Only one single testosterone measurement
					- Time of day for blood collection was not standardized; however, the effects of these varying time points of blood collection might be minimal due to less circadian fluctuation in testosterone levels in older men and no significant circadian variation of DHT levels [141]
	- Calculated free testosterone			- TT and cFT as well as SHBG were not significantly associated with all-cause mortality (HR 1.05 (0.88–1.25) for testosterone; HR 1.04 (0.88–1.23) for cFT)	
	- DHT				
	- Calculated free DHT				
Friedrich et al., 2012, [69]	- Total testosterone	Analysis from two German prospective cohort studies (DETECT and SHIP trial) (mean follow-up of 5.5 years)	3942 men	- Cox regression analyses adjusted for waist-to-height ratio, smoking and physical activity revealed that men with total serum testosterone levels below the 10th percentile showed an increased independent risk of all-cause mortality compared to subjects with higher hormone levels (HR 1.54, 95 % CI 1.20–1.99, *p* < 0.01); by additional inclusion of liver disease, increased blood pressure, diabetes and hsCRP as confounders the results were still significant for the 10th percentile as a cut-off (HR 1.38, 95 % CI 1.06–1.78, *p* = 0.02); using the 20th percentile as a cut-off resulted in no significant difference in all-cause morality between lower and higher testosterone levels (unadjusted model: HR 1.25, 95 % CI 0.99–1.57, *p* = 0.06)	- Only one single serum total testosterone measurement at baseline
					- Blood collection at different timepoints during the day; however, serum samples showed only minor differences in testosterone levels collected before and after noon; therefore the effect of the diurnal variation seems to be minimal
				- In univariate Kaplan-Meier survival analysis patients with lower serum total testosterone levels (cut-off: 10th percentile) showed significantly higher all-cause mortality compared to subjects with non-low hormone levels (*p* < 0.01)	- Use of different assay systems to determine total testosterone
					- No measurement of SHBG and thus no calculation of free testosterone levels
Grossmann et al., 2014, [70]	- Total testosterone	Single-center, prospective observational cohort study (median follow-up of 8.5-years)	221 patients with CKD III-IV, undergoing dialysis or KTR	- Cox proportional hazards regression showed significant independant association between low total testosterone levels and high mortality rates (*p* = 0.01)	- Small female sample size
					- Single-center study
	- DHT				- No data about patients with CKD I-II
	- Estrone			- Low testosterone concentrations were independent mortality predictors in men, whereas sex steroid levels in women showed no significant association with all-cause mortality	
	- Estradiol				
	- DHEA				
Araujo et al., 2007, [71]	- Total testosterone	Prospective population-based observational cohort study (secondary chart analysis of MMAS study) (15.3-year follow-up)	1686 men aged 40 to 70 years, population-based randomly sampled	- In multivariate, age-adjusted models TT levels as well as FT, DHT and SHBG were not significantly associated with all-cause mortality	- Inclusion of mostly white men of higher socioeconomic status; thus results may not be generalizable
	- Free testosterone			- Categorizing TT, FT, DHT and SHBG into 5 quintiles yielded no significant correlation with all-cause mortality either (for TT: reference group (RR = 1): TT ≥ 650 ng/dl; < 370 ng/dl: RR 1.24 (95 % CI 0.89–1.73); 370–466 ng/dl: RR 1.00 (95 % CI 0.70–1.42); 466–545 ng/dl: RR 1.05 (95 % CI 0.74–1.47); 545–650 ng/dl: RR 1.20 (95 % CI 0.86–1.69) (p for trend = 0.50))	- No data about women in this study
	- DHT				
	- SHBG				
				- Lowest quintile of free testosterone level was significantly associated with decreased IHD mortality (p for trend = 0.02) and increased respiratory disease mortality (p for trend = 0.002);	
				- In conclusion, TT and FT as well as DHT seem to have a relatively weak or no association with all-cause mortality	
De Padua Mansur et al., 2012, [72]	Estrone	Single-center, prospective observational cohort study (5.8 +/− 1.4-year follow-up)	251 postmenopausal women in the ambulatory care clinic of a tertiary cardiology hospital	- Kaplan Meier survival curve showed a significant association between higher all-cause mortality in women and low estrone levels (<15 pg/ml) (*p* = 0.039)	- Single-center study
					- No data about male patients
				- Multivariate Cox regression analysis adjusted for diabetes, body mass index, dyslipidemia, and family history showed estrone to be the only independent predictor for all-cause mortality (OR 0.45 (95 % CI 0.21–0.95); *p* = 0.038)	- Only 32 deaths and thus relatively low statistical power

*ACTH* adrenocorticotropic hormone, *APACHE II* Acute Physiology And Chronic Health Evaluation, *ApoE* apolipoprotein E, *AUC* area under the receiver operating characteristic curve, *CAD* coronary artery disease, *CAP* community-acquired pneumonia, *CAPNETZ* network of excellence community-acquired pneumonia, Germany, *cFDHT* calculated free dihydrotestosterone, *cFT* calculated free testosterone, *CHS* Cardiovascular Health Study, *CI* confidence interval, *CKD* chronic kidney disease, *COPD* chronic obstructive pulmonary disease, *CRP* C-reactive protein, *CURB-65 score* new-onset confusion urea >7 mmol/L respiratory rate ≥30 breaths per minute systolic or diastolic blood pressure <90 mmHg or ≤60 mmHg respectively age ≥65 years (pneumonia/LRTI risk scoring system), *CVD* cardiovascular disease, *DETECT* Diabetes Cardiovascular Risk-Evaluation: Targets and Essential Data for Commitment of Treatment, *DHEA* dehydroepiandrosterone, *DHEA-S* dehydroepiandrosterone-sulfate, *DHT* dihydrotestosterone, *FC* free cortisol, *FT* free testosterone, *HD* hemodialysis, *HR* hazard ratio, *hsCRP* high-sensitive C-reactive protein, *ICU* intensive care unit, *IHD* ischemic heart disease, *IL-1beta* interleukin-1 beta, *KTR* kidney transplant recipients, *LDL* low-density lipoprotein, *MMAS* Massachusetts Male Aging Study, *OR* odds ratio, *p* p-value are statistically significant at *p* < 0.05, *PSI* pneumonia severity index, *Q* quartile, *ROC* receiver operationg characteristic, *RR* relative risk, *sd-LDL* small dense low-density lipoprotein, *SHBG* sex hormone binding globuline, *SHIP* Study of Health in Pomerania, *TC* total cortisol, *TT* total testosterone, *VLDL* very-low-density lipoprotein

Conflicting results mark studies evaluating the role of serum DHEA as a mortality predictor. A prospective clinical trial conducted by Mueller et al. demonstrated that increased DHEA predicts 6-week mortality in CAP, whereas blood levels of DHEA-S as well as different ratios between cortisol and DHEA, cortisol and DHEA-S, and DHEA and DHEA-S shows no significant correlation with all-cause mortality [53]. Contrary to these findings, Kolditz et al*.* found no difference in DHEA levels in non-survivors compared to survivors, whereas the lack of significance for the ratio between DHEA and DHEA-S as a mortality predictor accords with the results of Mueller et al. [53, 61]. Data on patients suffering from sepsis reveal a noticeable dissociation between cortisol and DHEA in non-survivors compared to survivors, and from this observation it can be inferred that an increased cortisol/DHEA ratio is a prognostic marker in sepsis [64]. However, although the cortisol/DHEA ratio in clinical studies of CAP patients is not significant in the prediction of mortality, nevertheless, in non-survivors an increased cortisol/DHEA ratio occurs as a tendency [53]. In elderly Swedish men, an inverse association between serum DHEA and DHEA-S is evident, whereas low serum DHEA and high DHEA-S levels are independent predictors of all-cause mortality [65]. Furthermore Hsu et al. demonstrated a marked and independent association between low levels of plasma DHEA-S and higher mid-term all-cause mortality in men with chronic kidney disease (CKD) who were undergoing hemodialysis [66].

Table 1 depicts the strong association between increased serum estradiol and progesterone levels and 28-day mortality in patients suffering from pneumonia-related septic shock, whereas serum testosterone levels are not noticeably different between survivors and non-survivors after a 28-day follow-up [67]. A prospective clinical study of elderly men who were free of cardiovascular disease at the time of blood sample collection, revealed a significant inverse association between dehydrotestosterone (DHT) and calculated free DHT levels with all-cause mortality during a follow-up period of 9 years, whereas total testosterone and calculated free testosterone showed no significant correlation with mortality [68]. Contrary to these findings, Friedrich et al. and Grossmann et al. observed that low serum total testosterone levels in men with and without CKD significantly and independently predict all-cause mortality [69, 70], whereas another population-based clinical study of 40- to 70-year-old men [71] observed no significant association with either total or free testosterone, or with DHT in all-cause mortality in multivariate-adjusted analysis.

In postmenopausal women low estrone levels independently predict long-term all-cause mortality [72].

### Biogenic amines

#### I. L-arginine metabolites: Asymmetric dimethylarginine and symmetric dimethylarginine

##### Pathway and physiological role

Endothelial nitric oxide synthases (NOS) synthesize nitric oxide (NO) from L-arginine (Arg). NO is responsible for vasodilatation and acts as a neurotransmitter with several functions, including memory formation [73]. NO promotes relaxation of airway smooth muscle in the respiratory system, but NO deficiency results in hyperreactivity of the airway [74]. Asymmetric dimethylarginine (ADMA) and its isomer symmetric dimethylarginine (SDMA) are endogenous compounds of methylated protein turnover. ADMA—but not SDMA—is a competitive inhibitor of NOS and thus indirectly of NO [75]. One study shows that ADMA is present in the sputum of COPD patients [76].

##### Current research (Table 2)

A large community-based study of middle-aged participants revealed a positive association between ADMA levels and long-term mortality, whereas the Arg/ADMA ratio was inversely associated with death rate [77]. In people >65 years, ADMA is the strongest predictor of all-cause mortality, while some traditional risk factors—e.g., body mass index, systolic and diastolic blood pressure, male sex or triglycerides—lose their predictive power in this age group [78]. Additionally, elevated ADMA and SDMA serum levels correlate noticeably with higher all-cause mortality in patients with stable CHD [79] and in critically ill patients [80, 81]. Contrary to these findings, another study—after adjustment for NT-proBNP, hsCRP, troponin T and cardiorenal indices—reveaed that SDMA, but not ADMA, is an independent predictor of all-cause mortality [82].

**Table 2:** Summary of selected literature relevant to biogenic amines in all-cause mortality prediction

First author, year, reference	Marker	Study type	Study population	Key findings	Limitations
Böger et al., 2009, [77]	- ADMA	Observational, prospective cohort study (median follow-up of 10.9-year)	3,319 middle-aged participants (Framingham Offspring Study)	- ADMA was positively associated with mortality (multivariable-adjusted HR 1.21, 95 % CI 1.07–1.37, *p* = 0.003)	- Only middle-aged subjects
	- L-arginine				
				- Arginin/ADMA-ratio was inversely associated with mortality (HR 0.80, 95 % CI 0.69–0.93, *p* = 0.004)	
				- Higher ADMA levels (*p* = 0.0002) and lower Arg/ADMA-ratios (*p* = 0.0005) were associated with elevated mortality in non-diabetic subjects	
Pizzarelli et al., 2013, [78]	- ADMA	Single-center, prospective cohort study (median follow-up of 110 months)	1,025 randomly selected adults (>65 years) living in Chianti area, Tuscany, Italy	- Plasma ADMA was a strong predictor of all-cause mortality (HR 1.26, 95 % CI 1.10–1.44, *p* < 0.001) and there was a non-significant trend for cardiovascular mortality (HR 1.22, *p* = 0.07) after multivariate adjusment	- Single-center study
	- L-arginine				- Only one ethnic population
				- There was no association of ADMA with mortality in subjects with high L-arginine, but an increase in mortality in those with normal to low L-arginine	
Siegerink et al., 2013, [79]	- ADMA	Multicenter, observational, prospective cohort study (median follow-up of 8.1 years)	1,148 subjects suffering from myocardial infarction/ACS, or undergoing cardiac surgery due to CHD	- After adjustment for confounders higher levels of ADMA (HR 1.15, 95 % CI 0.95–1.37) and SDMA (HR 1.29, 95 % CI 1.09–1.52) were associated with an increase in all-cause mortality	- Selection bias
	- SDMA				- Only two-center study
			(KAROLA Study, Germany)		
Koch et al., 2013, [80]	ADMA	Single-center, observational prospective cohort study (3-year follow-up)	255 ICU patients and 78 healthy controls living in Germany	- ICU patients had higher serum ADMA levels than healthy controls (median 0.48 vs. 0.36 μmol/L, *p* < 0.001)	- Short-term intensive care (<72 h) patients were excluded
				- ICU non-survivors had higher ADMA levels compared with ICU survivors (median 0.62 vs. 0.44 μmol/L, *p* < 0.001)	- Single-center study
				- High ADMA levels predicted all-cause mortality in critically ill patients (*p* < 0.001)	
				- ADMA levels increased during 7 days of ICU therapy (*p* < 0.001)	
Koch et al., 2013, [81]	SDMA	Single-center, observational prospective cohort study	247 ICU patients and 84 healthy controls living in Germany	- ICU patients had higher serum SDMA levels than healthy controls (median 0.84 vs. 0.38 μmol/L, *p* < 0.001)	- Short-term intensive care (<72 h) excluded
				- ICU non-survivors had higher SDMA levels compared with ICU survivors (median 1.33 vs. 0.74 μmol/L, *p* = 0.001)	- Single-center study
		(3-year follow-up)		- High SDMA levels predicted poorer long-term prognosis in critically ill patients (*p* < 0.001)	
Gore et al., 2013, [82]	- SDMA	Observational prospective cohort study (median follow-up of 7.4 years)	3,523 adults aged 30 to 65 years (Dallas Heart Study)	- After adjustment for cardiorenal indices, age, sex, race, NT-proBNP, hsCRP and Troponin, SDMA, but not ADMA, was associated with all-cause mortality (HR 1.86, 95 % CI 1.04–3.30, *p* = 0.01)	- Single blood sampling
	- ADMA				
Suzuki et al., 2011, [85]	- Kynurenine	Single-center, observational prospective cohort study	129 Japanese patients with CAP and 64 healthy controls	- CAP patients had elevated levels of Kyn (*p* < 0.0001) and reduced levels of Trp (*p* < 0.0001) compared with healthy controls and thus higher Kyn/Trp ratios (*p* < 0.0001)	- Single-center study
	- Tryptophan				- Small sample size
					- No information about duration of follow-up
				- Increasing severity of sepsis and CAP (PSI and CURB-65 score) was associated with higher Kyn levels, lower Trp levels and higher Kyn/Trp ratios.	
				- Non-survivors had higher Kyn levels (*p* = 0.023) and lower Trp levels (*p* = 0.032) and as a result, higher Kyn/Trp ratios (*p* = 0.005)	
Darcy et al., 2011, [86]	- Kynurenine	Single-center, observational prospective cohort study	50 patients from Australia with severe sepsis (organ dysfunction or shock), 30 with non-severe sepsis and 40 hospital controls	- Sepsis patients had elevated levels of Kyn (*p* < 0.0001) and reduced levels of Trp (*p* < 0.0001) and thus higher Kyn/Trp ratios (*p* < 0.0001) compared with hospital controls.	- Single-center study
	- Tryptophan				- Small sample size
				- Kyn/Trp ratio was increased in severe sepsis compared with non-severe sepsis (*p* = 0.0006)	
		(28-day follow-up)			
				- Kyn/Trp ratio did not differ between survivors and non-survivors by day 28 of the study (*p* = 0.2)	
Huttunen et al., 2010, [87]	- Kynurenine	Single-center, observational prospective cohort study (30-day follow-up)	132 patients with bacteremia admitted to Tampere University Hospital in Finland	- Maximum Kyn/Trp ratios were significantly elevated in non-survivors (30-day case fatality) compared with survivors (193.7 vs. 82.4 μmol/mmol; *p* < 0.001)	- Small sample size
	- Tryptophan				- Single-center study
Qian et al., 2013, [92]	3-nitrotyrosine	Single-center, observational prospective cohort study (90-day follow-up)	158 patients with AKI, 12 critically ill patients without AKI, 15 healthy controls	- Patients with AKI had higher 3-NT/Tyr levels than healthy and critically ill controls (*p* < 0.001)	- Relatively small size study
					- Single-center study
				- High 3-NT/Tyr was associated with higher 90-day mortality (*p* = 0.025)	

*3-NT* 3-nitrotyrosine, *ACS* acute coronary syndrome, *ADMA* asymmetric dimethylarginine, *AKI* acute kidney injury, *CAP* community-acquired pneumonia, *CHD* coronary heart disease, *CI* confidence interval, *CURB-65 score* new-onset confusion, urea >7 mmol/L, respiratory rate ≥30 breaths per minute, systolic or diastolic blood pressure <90 mmHg or ≤60 mmHg, respectively, age ≥65 years (pneumonia/LRTI risk scoring system), *HR* hazard ratio, *hsCRP* high-sensitivity C-reactive protein, *ICU* intensive care unit, *KAROLA* Langzeitfolge der KARdiOLogischen Anschlussheilbehandlung, Germany, *Kyn* kynurenine, *NT-proBNP* pro-B-type natriuretic peptide, *p* p-value are statistically significant at *p* < 0.05, *PSI* pneumonia severity index, *SDMA* symmetric dimethylarginine, *Trp* tryptophan, *Tyr* tyrosine

#### II. Kynurenine

##### Pathway and physiological role

Tryptophan (Trp) is an essential amino acid. Degradation of Trp to its toxic metabolite kynurenine (Kyn) is catalyzed by the enzyme indoleamine 2,3-dioxygenase (IDO), which is expressed in a variety of cells; e.g., monocyte-derived macrophages and dentritic cells [83]. Moreover, IDO induces inhibition of T-cell proliferation [84] and its activity is measured by the kynurenine-to-tryptohan ratio (Kyn/Trp ratio) [85–87].

##### Current research (Table 2)

Subjects with CAP [85] and sepsis [86] show significantly higher Kyn levels and lower Trp levels compared to controls. As a result, the Kyn/Trp ratio, which represents the IDO activity, is elevated in CAP and sepsis [85, 86]. Increased severity of CAP (defined by PSI and CURB-65 score) [85] and of sepsis [86] are associated with higher Kyn levels, higher Kyn/Trp ratios and lower Trp levels. In patients with CAP [85] or bacteremia [87], short-term non-survivors have higher Kyn/Trp ratios than do survivors, whereas the sepsis group manifests no marked difference in Kyn/Trp ratio between non-survivors and survivors in the short-term [86].

#### III. Nitrotyrosine

##### Pathway and physiological role

Homeostatic balance is usually maintained between the formation of reactive oxygen species (ROS) and its removal by endogenous antioxidants. Oxidative stress arises from an imbalance towards ROS production [88]. Peroxynitrite (ONOO^−^) is a potent oxidant of this ROS group and catalyzes nitration of the non-essential amino acid tyrosine (Tyr) to the non-proteinogenic amino acid 3-nitrotyrosine (3-NT) [89]. Accordingly, nitrotyrosine is a characteristic marker of oxidative stress (e.g., inflammation) [90].

Protein-associated nitrotyrosine is lower in smokers than in non-smokers, evidence consistent with a lower production of endothelial NO in cigarette smokers. In contrast, smokers with COPD show higher average nitrotyrosine levels in plasma proteins compared with smokers without COPD. Therefore, it seems that chronic inflammatory processes in COPD patients increase the nitration of Tyr [91].

##### Current research (Table 2)

As shown in Table 2, subjects with hospital-acquired acute kidney injury (AKI) have higher 3-nitrotyrosin/tyrosine ratios (3-NT/Tyr) than do critically ill subjects without AKI and healthy controls. In the AKI group, the 3-NT/Tyr ratio is positively associated with 90-day mortality independent of the severity of illness [92].

#### Trimethylamine-N-oxide, betaine, choline

##### Pathway and physiological role

Synthesis of phospholipids to build cell membranes, acetylcholine for neurotransmission just as the methyl group metabolism depend on dietary phosphatidylcholine (PC)/choline intake [93]. The liver and kidney can both oxidize choline to betaine. Betaine is an important metabolite in methyl group metabolism; it functions as a methyl group donor to form methionine [94, 95]. Eggs, red meat, beef and chicken liver, wheat germ, bacon, dried soy beans and pork contain the highest concentration of total choline. Betaine levels are high in wheat bread, wheat bran, wheat germ, spinach and shrimp [96]. Intestinal microbes (gut microflora) transform choline and other quaternary ammonium compounds (e.g., betaine) to the gas trimethylamine (TMA) [97]. This gas is reabsorbed efficiently into the circulation and then into human liver. Hepatic flavin-containing monooxygenases (FMOs) rapidly oxidize TMA to trimethylamine-*N*-oxide (TMAO) [98]. Thus TMA is not produced via intermediary metabolism; rather, it depends on intestinal microbial breakdown of choline and other precursors. Thus for the production of TMA and its oxidation product TMAO, intact and functional intestinal microflora are essential [97, 99].

##### Current research (Table 3)

Several animal studies show the effects of elevated TMAO levels. An increase in the concentration of plasma TMAO was observed in mice, which were fed with either choline or TMAO [99, 100]. After 16 weeks of feeding there was a marked increase in tubulointerstitial fibrosis and serum cystatin C [100]. A significant positive correlation between plasma levels of TMAO and atherosclerotic plaque size after 20 weeks of feeding in both male and female mice was observed. Furthermore, macrophages from mice supplemented with either choline, betaine or TMAO showed augmented lipid-loaded macrophages and higher levels of the macrophage scavenger receptors CD36 and SR-A1. These receptors implicate atherosclerosis. In a mouse model, application of broad-spectrum antibiotics for 3 weeks suppressed dietary choline-induced formation of macrophage foam cells and choline-mediated enhancement in atherosclerosis [99].

**Table 3:** Summary of selected literature relevant to trimethylamine-N-oxide in all-cause mortality prediction

First author, year, reference	Marker	Study type	Study population	Key findings	Limitations
Tang et al.., 2015, [102]	TMAO	Single-center, prospective observational cohort-study (5-year follow-up)	112 adults with stable but symptomatic chronic systolic HF (left ventricular ejection fraction ≤35 %) (Cleveland Clinic)	- After adjustment for age, eGFR, and NT-proBNP levels, higher TMAO levels were associated with poor prognosis (death/transplantation) (HR 1.46; 95 % CI 1.03-2.14; *p* = 0.03)	- Single-center study
					- Selection bias
				- TMAO levels were higher in subjects with higher plasma NT-proBNP levels and NYHA functional class III or IV (*p* = 0.02)	
Tang et al.,, 2013, [103]	TMAO	Single-center, prospective interventional study (9-day follow-up)	40 healthy adults without chronic illnesses, active Infections or antibiotic therapy (Cleveland Clinic)	- Increasing plasma levels of TMAO after oral phosphatidylcholine challenge	- Small study population
					- Only healthy adults included
				- In 6 adults, plasma levels of TMAO were markedly suppressed after a weekly therapy with broad-spectrum antibiotics and reappeared after withdrawal of antibiotics.	
Tang et al., 2013, [103]	TMAO	Single-center, prospective observational cohort-study (3-year follow-up)	4,007 adults undergoing elective diagnostic cardiac catheterization without evidence of an ACS (Cleveland Clinic)	- Elevated plasma levels of TMAO were associated with a higher risk of a major cardiovascular event after adjustment for traditional risk factors (*p* < 0.001)	- Single-center study
					- Selection bias
				- Prognostic value of elevated plasma levels of TMAO remained significant in low-risk subgroups	
Tang et al.., 2014, [101]	TMAO	Single-center, prospective observational cohort study (5-year follow-up)	720 subjects with a history of HF (Cleveland Clinic)	- Subjects with HF (5.0 μmol) had higher median TMAO levels than subjects without HF (3.5 μmol; *p* < 0.001)	- Single-center study
					- Selection bias
				- TMAO levels were predictive of 5-year mortality risk after adjustments for traditional risk factors, BNP levels and eGFR (HR 1.75; 95 % CI 1.07–2.86; *p* < 0.001)	
Tang et al.., 2015, [100]	TMAO	Single-center, prospective observational cohort-study (5-year follow-up)	521 subjects with CKD (eGFR < 60 mL/min) and 3,166 non-CKD subjects (Cleveland Clinic)	- TMAO levels were increased in CKD subjects (median, 7.9 μmol/L) compared with non-CKD subjects (median, 3.4 μmol/L), *p* < 0.001	- No specific results for CKD stage 1-2
					- Single-center study
				- Higher TMAO levels (quartiles 4 versus 1) were associated with an increase in 5-year all-cause mortality in CKD subjects (HR 1.93; 95 % CI 1.13–3.29; *p* < 0.05) and non-CKD subjects after adjustment for traditional CVD risk factors and eGFR (HR 1.47; 95 % CI 1.02–3.29; *p* < 0.05)	

*ACS* acute coronary syndrome, *BNP* brain natriuretic peptide, *CI* confidence interval, *CKD* chronic kidney disease, *CVD* cardiovascular disease, *eGFR* estimated glomerular filtration rate, *HF* heart failure, *HR* hazard ratio, *NT-proBNP* pro-B-type natriuretic peptide, *NYHA* New York Heart Association functional classification, *p* p-value are statistically significant at *p* < 0.05; *TMAO* trimethylamine-*N*-oxide

In humans (3), higher plasma NT-proBNP [101, 102] and advanced left ventricular diastolic dysfunction are associated with elevated plasma betaine, choline and TMAO. TMAO levels are markedly higher in patients with heart failure NYHA III/IV or diabetes mellitus. Baseline plasma levels of TMAO have marked prognostic value for major adverse cardiovascular events in low-risk subgroups after adjustments for age and cardiorenal indices [102, 103]. Since cardiovascular disease (CVD) remains the major cause of deaths [104], these findings might be predictive of poor outcome. In addition, elevated TMAO levels are predictive of 5-year mortality risk in subjects with heart failure after adjustment for cardiovascular risk factors and renal function [101].

In healthy adults a dietary PC challenge raises TMAO level. In six human participants this elevation was suppressed with oral broad-spectrum antibiotics [103].

A high correlation is evident between plasma and urine levels of TMAO, which indicates an effective urinary clearance of TMAO [103]. Median TMAO blood levels in subjects with CKD stage 3 or beyond are markedly higher than in non-CKD subjects. Moreover, there is a modest correlation between elevated TMAO with low estimated glomerular filtration rate (eGFR) and high cystatin C. After adjustment for traditional cardiovascular risk factors an increased all-cause mortality rate at 5 years for CKD subjects with a higher TMAO level is evident. In subjects with preserved eGFR (>60 mL/min per 1.73 m^2^) higher TMAO levels are associated with higher 5-year mortality, particularly in patients who have concomitant high cystatin C levels [100].

### Intermediate energy metabolism

#### Lactate

##### Pathway and physiological role

Glycolysis produces the metabolite pyruvate. Lactate dehydrogenase (LDH) converts pyruvate to lactic acid under anaerobic conditions. Lactate acid dissociates spontaneously to lactate and H^+^ in aqueous solutions. Thus lactate is the end product of anaerobic glucose metabolism in erythrocytes, perivenous hepatocytes, skeletal myocytes and skin cells. A normal plasma lactate concentration is 0.3-1.3 mmol/L and the liver removes 70 % of the lactate. An imbalance between systemic oxygen demand and oxygen availability (e.g., hypoperfusion in patients with sepsis) heightens anaerobic metabolism and results in lactic acidosis [105, 106].

##### Current research (Table 4)

In children with pneumonia, relative risk for in-hospital mortality is higher if the lactate level is above 2 mmol/L [107]. In adults with pneumonia, lactate level is the better predictor of 28-day mortality than the CURB-65 score, whereas a combination of CURB-65 with lactate level improves the predictive value of CURB-65 alone (Table 4) [108].

**Table 4:** Summary of selected literature relevant to intermediate energy metabolism in all-cause mortality prediction

First author, year, reference	Marker	Study type	Study population	Key findings	Limitations
Ramakrishna et al., 2012, [107]	Lactate	Single-center, observational prospective cohort-study (follow-up until hospital discharge)	233 Malawian children with pneumonia	- The odds ratio for in-hospital mortality (25 deaths) in children with lactate >2 mmol/L was 7.48 (95 % CI 1.72–32.6) compared with children with lactate <2 mmol/L	- Single-center study
					- Low statistical power (only 25 deaths)
Chen et al., 2015, [108]	Lactate	Single-center, observational prospective cohort-study (28-day follow-up)	1,641 patients with pneumonia (861 inpatients, 780 outpatients) (Emergency Department of Beijing Chao-Yang Hospital)	- Non-survivors had higher lactate and CURB-65 scores compared with survivors (*p* < 0.001)	- Single-center study
					- No information about microorganisms
				- Lactate predicted 28-day mortality better than CURB-65 score (AUC 0.823 vs. 0.692; *p* < 0.01)	
				- Combination of lactate and CURB-65 score improved the predictive value of CURB-65 score alone (AUC 0.851)	

*AUC* area under the receiver operating characteristic curve, *CI* confidence interval, *CURB-65 score* new-onset confusion, urea >7 mmol/L, respiratory rate ≥30 breaths per minute, systolic or diastolic blood pressure <90 mmHg or ≤60 mmHg, respectively, age ≥65 years (pneumonia/LRTI risk scoring system), *p* p-value are statistically significant at *p* < 0.05

Lactate >3.5 mmol/L in combination with a P_a_O_2_/FiO_2_ (PF) ratio <170 is associated with a higher 28-day mortality rate; accordingly, a combination of serum lactate and the PF ratio may be a useful predictor of mortality in patients with sepsis, including pneumonia [109]. Metabolic profiling of plasma from 90 critically ill intensive care unit (ICU) patients revealed significantly higher lactate levels in non-survivors compared with survivors in 28-day mortality [110].

#### Monosaccharides

##### Pathway and physiological role

Monosaccharides are the simplest form of sugar and therefore the most basic units of carbohydrates; they are important as an energy resource. The most important monosaccharides in human metabolism are the three aldohexoses (D-glucose, D-mannose, and D-galactose), and D-fructose, a sugar monomer of the ketohexose series of carbohydrates [111].

##### Current research (Table 5)

In patients with CAP (Table 5) , hypoglycemia [112] and hyperglycemia are associated with a higher rate of in-hospital mortality [112, 113] and in-hospital complications [113]. Furthermore, hyperglycemia is a predictor of death at 28 and 90 days after admission to hospital in patients with CAP and no pre-existing diabetes [114].

**Table 5:** Summary of selected literature relevant to monosaccharides in all-cause mortality prediction

First author, year, reference	Marker	Study type	Study population	Key findings	Limitations
Foltran et al., 2013, [112]	Glucose	Single-center, retrospective case-control study	1,018 Italian non-intensive care patients with pneumonia	- Plasma glucose levels of mean 86 mg/dl (95 % CI, 61–102 mg/dl) were associated with minimal risk of in-hospital mortality	- Only admission glucose levels
					- Single-center study
					- No information on important confounders (e.g. diabetes)
				- The OR was 1.33 (95 % CI 1.07–1.66) for each 10 mg/dl of increase in plasma glucose in hyperglycemic patients (>86 mg/dl)	
McAlister et al., 2005, [113]	Glucose	Multicenter, observational prospective cohort study (median length of in-hospital stay was 6 days, follow-up until hospital discharge)	2,471 Canadian adults with CAP	- Patients with an admission glucose level >11 mmol/l had an increased risk of death (13 vs. 9 %, *p* = 0.03) and in-hospital complications (29 vs. 22 %, *p* = 0.01) compared to those with glucose levels <11 mmol/l	- Glucose only measured once
					- No examination of antiglycemic treatments
					- Not measured long-term glucose (HbA1c)
				- Patients with admission glucose >11 mmol/l had 73 % higher mortality (95 % CI 12–108 %) and a 52 % higher (12–108 %) risk of in-hospital complications compared to patients with glucose levels ≤6.1 mmol/l	
Lepper et al., 2012, [114]	Glucose	Multicenter, prospective cohort study (6-month follow-up)	6,891 German patients with CAP (CAPNETZ Study)	- In patients with no pre-existing diabetes an increased serum glucose level was a predictor of death at 28 and 90 days	- Treatment of pneumonia was left to the discretion of the doctor
				- Mild hyperglycemia (6-10.99 mmol/l) on admission was associated with an increased risk of death at 90 days (HR 1.56, 95 % CI 1.22–2.01, *p* < 0.001), and this risk increased to 2.37 (95 % CI, 1.62–3.46, *p* < 0.001) when serum glucose levels were ≥14 mmol/l	- Did not examine changes in serum glucose
					- Glucocorticoid treatments were not recorded
					- No HbA1c tests

*CAP* community-acquired pneumonia, *CAPNETZ* network of excellence community-acquired pneumonia, Germany, *CI* confidence interval, *HbA1c* glycated hemoglobin, *HR* hazard ratio, *OR* odds ratio, *p,* p-value are statistically significant at *p* < 0.05

#### Oxidative status

##### Pathway and physiological role

Reactive oxygen species (ROS) and reactive nitrogen species (RNS) are important in regulating the proliferation of cells and their survival. However, a sudden and prolonged surge of ROS and RNS may contribute to cell death. The redox homeostasis of cells, which maintains a balance between the generation and elimination of ROS and RNS ensures that endogenous and exogenous stimuli are modulated. Altered redox homeostasis leads to oxidative stress, which may cause aberrant cell death and the development of disease [115]. Glutathione (gamma-glutamyl-cysteinyl-glycine, GSH) is the predominant antioxidant non-protein cysteine-containing thiol, which is found in all animal cells. It is important in maintaining the status of cellular redox, which is determined by the ratio of the concentration of oxidizing equivalents to reducing equivalents [116], and it is the principal intracellular defense against oxidative stress. GSH has two redox forms: reduced GSH and glutathione disulfide (GSSG, the oxidized form) [117]. Cells can excrete GSSG or restore it to GSH through the action of GSH reductase. *De novo* synthesis of GSH from its amino-acid constituents—a process that involves two enzymatic steps catalyzed by glutamate cysteine ligase and GSH synthetase— [118] is essential for the adaptive elevation of GSH during oxidative stress [117]. The rate-limiting step in the *de novo* synthesis of GSH occurs at the cellular level of the amino acid cysteine [119]. Additionally, reduction of cystine to cysteine is an important mechanism for intracellular GSH elevation *in vivo* in lungs [120]. During oxidative stress and inflammation, synthesis of GSH is upregulated, possibly providing a protective or adaptive response to subsequent oxidative and inflammatory stress. An imbalance of antioxidant and proinflammatory genes can lead to chronic inflammation [117].

Homocysteine is built from the breakdown of the essential amino acid methionine and can subsequently be converted to cystathionine with vitamin B6 and further to cysteine. Conversely, vitamin B12 promotes remethylation of homocysteine to methionine [121, 122]. The latter reaction is catalyzed by the enzyme methionine synthase, requiring 5-methyltetrahydrofolate, the main circulating form of folate, and vitamin B12 in its co-factor form methylcobalamin [122].

##### Current research (Table 6)

Table 6 outlines a clinical study that ascertained the status of glutathione—reflected by the reduced ratio of glutathione (GSH) to total glutathione in whole blood—as an independent mid-term predictor of all-cause mortality in post-ICU patients, whereas the total concentration of glutathione as well as the concentration of GSH alone revealed no association with 6-month mortality [118]. Although total glutathione and GSH concentrations in ICU patients have been observed as low, the quartile with the highest redox ratio of glutathione (GSH/total glutathione) carries the highest post-ICU all-cause mortality risk [118]. Lower levels of glutathione-S-bimane (GSB)—that is, intracellular GSH measured by fluorescence-activated cell sorter (FACS)—in CD4 T cells are prominent long-term mortality predictors in HIV-infected individuals, independent of CD4 T cell count [123].

**Table 6:** Summary of selected literature relevant to oxidative status in all-cause mortality prediction

First author, year, reference	Marker	Study type	Study population	Key findings	Limitations
Rodas et al., 2012, [118]	- Total glutathione	Single-center, prospective observational cohort study (6-month follow-up)	174 patients admitted to the ICU (without thoracic, neurosurgery and trauma cases) (>17 years of age)	- After division of patients into quartiles according to total concentration of glutathione and GSH in whole blood, no relationship to mortality was observed (data not shown); division of patients into quartiles according to GSH/total glutathione ratio showed a significant higher 6-month-all-cause mortality for the quartile with the highest ratio compared to the lower three quartiles (*p* = 0.026); the AUC of GSH/total glutathione ratio regarding all-cause mortality prediction was 0.56 (*p* = 0.18) with an optimal cut-off for the GSH/total glutathione ratio of 0.66; a stepwise multiple logistic regression analysis regarding 6-month-mortality prediction showed an OR of 2.35 (CI 1.02-5.41; *p* < 0.001) for the GSH/total glutathione ratio	- The study only represented the group actually studied, namely patients >17 years of age admitted to the ICU
	- GSH				
	- Glutathione redox status (GSH/total glutathione ratio)				- Observational studies allow no statement about causal association
					- Redox status of glutathione in plasma might not be representative for the intracellular status (Nevertheless, the plasma ratio of GSH/total glutathione could be useful as a biomarker to predict post-ICU mortality)
Herzenberg et al., 1997, [123]	- Total glutathione	Single-center, prospective observational cohort-study (2-3-year follow-up)	204 HIV-infected patients, who were screened for enrollment into a clinical trial designed to determine whether orally NAC replenishes GSH in subjects with low GSH levels; however, subjects who were finally enrolled in the NAC trial were not included in the present observational cohort study	- Logistic regression analyses demonstrated an association between increased baseline GSB and improved 2-3-year survival, whereas baseline GSB levels below normal values showed a higher mortality (*p* < 0.0001)	- Selection bias due to exclusion of patients who finally were trial subjects of the NAC interventional trial
	- GSH				
	- GSB				
					- No data about plasma glutathione
				- Kaplan-Meier survival curves demonstrated that patients with baseline GSB levels below 1.05 show a significantly increased 2-3-year all-cause mortality compared to subjects with higher baseline GSB levels (*p* = 0.005); the above named optimal treshold of 1.05 for separating survivors from non-survivors was determined using ROC analyses for 2-3-year survival	
				- In proportional hazard analyses with inclusion of GSB levels as well as CD4 T cell counts in the model, GSB has still been demonstrated to be a significant, independent mortality predictor (RR 1.6 [95 % CI 1.1–2.5], *p* = 0.009), despite its significant correlation with CD4 T cell counts (*p* < 0.0001)	
Xiu et al., 2012, [124]	Homocysteine	Prospective observational cohort-study (up to 10-year follow-up)	1,412 elderly >65 years of age, selected from the Elderly Nutrition and Health Survey in Taiwan (NAHSIT) [142]	- In a model adjusted for sociodemographic, behavioral, and nutritional variables, plasma homocysteine levels >14.5 umol/l were associated with significantly higher mortality compared to those <9.3 umol/l (HR 1.8, 95 % CI 1.20–2.71; *p* = 0.002)	- Although adjustment for several known associations with homocysteine status has been undertaken, residual confounding is still possible
Wong et al. 2013, [125]	Homocysteine	Prospective observational population-based cohort study (follow-up of 5.1 +/− 1.3 years)	4,248 community-dwelling Australian men aged 70–88 years, selected from the Health in Men Study (HIMS) [143]	- After adjustment for frailty, age, education, living circumstances, smoking, cardiovascular disease, cardiovascular risk factors and renal function, high total plasma homocysteine levels (≥15 umol/l) were a significant all-cause mortality predictor (HR 1.25, 95 % CI 1.06–1.48, *p* < 0.05)	- Self-selection of study participants might have biased the findings toward lower homocysteine, lower age and fewer comorbidities compared to the non-respondents, which limits generalizability of study results and might have moved the results toward the null hypothesis with underestimation of associations
Swart et al., 2011, [126]	Homocysteine	Sub-study of a prospective observational population-based cohort study (11-year follow-up)	1,117 independently living elderly (mean age = 75.1 years), selected from the Longitudinal Aging Study Amsterdam (LASA)	- After adjustment for several confounders, women in the third and the fourth quartile of plasma homocysteine levels were associated with a significantly higher all-cause mortality risk compared to those in the first quartile (Q3 HR 1.70, 95 % CI 1.08–2.65; Q4 HR 1.91, 95 % CI 1.22–3.00), independently of vitamin B12 status; in men there was no significant difference in all-cause mortality rates between different quartiles of homocysteine levels in adjusted models	- Possible underestimation of the actual relationship between homocysteine levels and mortality due to selection bias, since excluded subjects were older, more likely to be cognitively impared, less physically active and had suffered CVD more often at baseline
Drewes et al., 2014, [127]	Homocysteine	Post-hoc subanalysis of the double-blind, randomized placebo-controlled study, stratified by plasma homocysteine levels (mean follow-up of 3.2 years)	3,522 subjects with history of or risk factors for CVD (aged 70–82), selected from the primary care setting in two of the three PROSPER study sites (Netherlands and Scotland)	- In the placebo group subjects with plasma homocysteine levels in the highest tertile showed a significantly higher all-cause mortality risk (HR 1.7, 95 % CI 1.2–2.5, *p* = 0.003) as well as a greater risk of fatal and nonfatal CHD (HR 1.8, 95 % CI 1.2–2.5, *p* = 0.001) compared to those with low plasma homocysteine levels	- Study not originally designed to collect blood samples for plasma homocysteine level assessment, which is why data could only be used from two of the three PROSPER study sites
					- Some blood samples have possibly been stored at room temperature for up to 8 h, which could have contributed to artificially high plasma homocysteine levels, and thus misclassification
				- Regarding all-cause mortality the pravastatin treatment group showed an absolute risk reduction of 4.6 % (95 % CI 0.78–8.4 %) in the subgroup with high homocysteine levels compared to -0.66 % (95 % CI -4.0–2.7) in the subgroup with low homocysteine levels (absolute risk reduction difference of 5.2 %, 95 % CI 0.19–10.3; *p* = 0.04)	
					- No data about the intakte of vitamin B, which may lower homocysteine levels
Waskiewicz et al., 2012, [128]	Homocysteine	Prospective observational, population-based cohort study (mean follow-up of 5.4 years)	7,165 Polish people aged 20–74 years	- In multivariable proportional hazards models adjusted for sex, age and cardiovascular risk factors, RR of all-cause long-term mortality was significantly higher in plasma homocysteine levels >10.51 umol/l compared to those <8.20 umol/l (HR 1.766, 95 % CI 1.197–2.605)	- The immunoenzymatic method used to determine homocysteine levels in this study has shown the lowest precision of measurements in comparative analyses of various homocysteine assay methods
				- In Kaplan-Meier survival curves patients with homocysteine concentrations in the highest tercile (>10.51 umol/l) showed significantly increased long-term all-cause mortality compared to subjects with homocysteine levels in the lowest tercile (*p* = 0.0003)	- Impossible to perform analyses of mortality related to stroke and ischemic heart disease due to low number of deaths in these groups
Naess et al., 2013, [129]	Homocysteine	Prospective, observational population-based cohort study (mean follow-up of 12.4 years)	198 patients with first ischemic stroke living in Hordaland County (mean age of 47.8 years)	- After adjustment for age, sex and CRP levels, Cox regression analysis – excluding patients with stroke caused by dissection – showed high plasma homocysteine levels (>9 ug/l) to be significantly and independently associated with all-cause mortality (HR 1.04, *p* = 0.02)	- Small sample size
					- Possible selection bias due to retrospective patient recruitment
					- High plasma homocysteine levels have been shown to be significant all-cause mortality predictors only after exclusion of patients with dissection, suggesting homocysteine to be a potential confounder in mortality prediction
Vieira et al., 2010, [130]	Homocysteine	Single-center, prospective observational cohort-study (2-year follow-up)	95 predialysis patients with chronic kidney disease (mean age of 69.4 years)	- Kaplan-Meier survival curves demonstrated a significant lower survival in patients with total plasma homocysteine levels and nutritional status assessment (by mSGA) above the mean level compared to lower levels (*p* = 0.04; survival at 24 months = 50 %)	- Small sample size
Tekin et al., 2012, [144]	Homocysteine	Single-center, prospective observational cohort-study (1-year follow-up)	70 patients with HF (left ventricle ejection fractions < 35 %) (mean age 60 +/− 12)	- Serum homocysteine levels were significantly higher in non-survivors compared with survivors (20.8 +/− 5.8 vs. 16.9 +/− 5.1 umol/l, *p* = 0.029)	- Small sample size
				- With an optimal cut-off value of >17.45 umol/l, the AUC of serum homocysteine levels with regard to mid-term mortality prediction was 0.855 (95 % CI 0.792–0.965, *p* < 0.001)	

*AUC* area under the receiver operating characteristic curve, *CI* confidence interval, *CHD* coronary heart disease, *CKD* chronic kidney disease, *CRP* C-reactive protein, *CVD* cardiovascular disease, *GSB* glutathione-S-bimane, *GSH* glutathione, *HF* heart failure, *HIMS* Health in Men Study, *HIV* human immunodeficiency virus, *HR* hazard ratio, *ICU* intensive care unit, *LASA* Longitudinal Aging Study Amsterdam, *mSGA* modified Subjective Global Nutritional Assessment, *NAC* N-acetylcysteine, *NAHSIT* Nutrition and Health Survey in Taiwan, *OR* odds ratio, *p* p-value are statistically significant at *p* < 0.05, *PROSPER* PROspective Study of Pravastatin in the Elderly at Risk, *Q* quartile, *ROC* receiver operationg characteristic, *RR* relative risk

Several clinical studies have demonstrated that elevated levels of plasma homocysteine independently predict long-term all-cause mortality in the elderly [124–127] as well as in younger adults [128, 129]. In contrast, in patients after ischemic stroke a marked association is evident between high plasma homocysteine and overall mortality only after exclusion of patients with dissection [129]. Another study showed a correlation between higher homocysteine levels and increased long-term mortality in the subgroup of patients with malnutrition and inflammation [130]. Additionally, elevated plasma homocysteine levels are discernibly related to a higher death rate only in older women [126]. Besides their relation to all-cause mortality, high homocysteine concentrations are independently associated with cardiovascular mortality [128].

#### Glycerophospholipids

##### Pathway and physiological role

Glycerophospholipids are glycerol-based phospholipids that form the bilayer in biological membranes. Their basic structure is composed of glycerol, to which a phosphoric acid and two fatty acids are attached as esters. An additional alcohol group (e.g., serine, choline, ethanolamine or inositol) attached to the phosphate allows the formation of distinct phosphoglycerides. The five classes of glycerophospholipids are phosphatidylcholine (PC, lecithin), phosphatidylethanolamine (PE), phosphatidylserine (PS), phosphatidylinositol (PI) and diphosphatidylgylcerol (cardiolipin) [131].

##### Current research (Table 7)

In lipid profiling of patients undergoing coronary angiography, 19 PC and 3 lysophosphatidylcholine (lysoPC) species reveal a noticeable association with mortality due to total and coronary artery disease. Ten of 19 PC and all 3 analyzed lysoPC species are inversely associated with mortality, whereas PC 32:0 reveals the strongest positive correlation with mortality [132]. Four lysoPC species—C16:0, C18:0, C18:1, and C18:2—account for over 90 % of total plasma lysoPC concentration. The levels of these main lysoPC species as well as total lysoPC concentrations are markedly reduced in sepsis, including pulmonary infections, compared to healthy controls (Table 7). Moreover, 30-day non-survivors show higher ratios of lysoPC/PC compared with survivors [133].

**Table 7:** Summary of selected literature relevant to sphingolipids and glycerophospholipids in all-cause mortality prediction

First author, year, reference	Marker	Study type	Study population	Key findings	Limitations
Sigruener et al., 2014, [132]	- Sphingomyelin	Single-center, prospective observational cohort-study (median follow-up of 8 year)	2583 CAD-positve patients and 733 controls (LURIC Study)	- 9 PC species (PC 30:0, 30:1, 32:0, 32:1, 34:1, 34:2, 36:1, 38:0, 38:2) were positively associated with mortality	- Single-center study
	- Phosphatidyl-choline				- No information about time of blood sampling
	- Lysophosphati-dylcholine			- PC 32:0 revealed the strongest positive association with mortality	
				- 10 PC species (PC 38:3–38:7, 36:4, 36:5, 40:6, 40:7) were significantly associated with a protective effect	
				- LysoPC 16:0, 18:0, and 18:2 were all associated with a protective effect	
				- The 4 SM species 16:0, 16:1, 24:1, and 24:2 showed positive association with mortality	
Drobnik et al., 2003, [133]	Lysophosphatidyl-choline	Single-center, prospective observational cohort-study (30-day follow-up)	102 patients with sepsis admitted to the University Hospital of Regensburg (Germany) and 56 healthy controls	- All 12 different lysoPC species and lysoPC-PC ratios were markedly decreased in patients with sepsis compared with healty controls	- Single-center study
					- Small patient populations
					- No information about causes of sepsis
				- The 4 lysoPC species, C16:0, C:18:0, C18:1, and C18:2, accounted for over 90 % of total lysoPC concentration	
Schlitt et al., 2006, [135]	Sphingomyelin	Multicenter, prospective observational cohort-study (median follow-up of 6 year)	1102 patients with CAD and 444 healthy controls	- CAD patients showed higher plasma SM levels than healthy controls (mean 51.8 vs. 44.9 mg/dl; *p* < 0.001)	- Only two-center study
					- No autopsy performed
				- In multivariate analysis, elevated SM (>48.1 mg/dl) was related to cardiovascular death or nonfatal myocardial infarction (HR 1.8, 95 % CI 1.0–3.3, *p* < 0.05) in patients with ACS	
Jiang et al., 2000, [136]	Sphingomyelin	Multicenter, observational case-control study	556 patients scheduled for coronary angiography in New York	- Patients with CAD had higher plasma SM concentrations compared with non-CAD patients (median 52 vs. 44 mg/dl, *p* < 0.0001)	- Only two-center study
					- No prospective design
				- The odds ratios for CAD patients for the third (2.83, 95 % CI 1.74–4.60, *p* < 0.0001) and fourth (2.59, 95 % CI 1.60–4.19, *p* = 0.0001) quartiles were higher than the first quartile	

*ACS* acute coronary syndrome, *CAD* coronary artery disease, *CI* confidence interval, *HR* hazard ratio, *LURIC* Ludwigshafen Risk and Cardiovascular Health study, Germany, *lysoPC* lysophosphatidylcholine, *OR* odds ratio, *p* p-value are statistically significant at *p* < 0.05, *PC* phosphatidylcholine, *SM* sphingomyelin

**Table 8:** Summary of selected literature relevant to acylcarnitines in all-cause mortality prediction

First author, year, reference	Marker	Study type	Study population	Key findings	Limitations
Kalim et al., 2013, [138]	Oleoylcarnitine	2 independent, observational case-control studies (ArMORR Study)	I: 100 non-survivors of 1-year HD and 100 survivors of at least 1-year HD matched for age, sex, and race	I: Oleoylcarnitine showed the strongest association with cardiovascular mortality after multivariable adjustment (OR ratio per SD 2.3, 95 % CI 1.4–3.8, *p* = 0.001)	- Measurement of basline metabolites after starting hemodialysis
					- Accuracy of ICD-9 codes for cardiac diagnoses not completely sensitive or specific
				II: Oleoylcarnitine was associated with cardiovascular death (OR per SD 1.4, 95 % CI 1.1–1.9, *p* = 0.008)	
			II: 100 non-survivors of 1-year HD and 200 survivors of at least 1 year HD		

*ArMORR* The Accelerated Mortality on Renal Replacement Study, United States, *CI* confidence interval, *HD* hemodialysis, *ICD-9* International Classification of Diseases, *OR* odds ratio, *p* p-value are statistically significant at *p* < 0.05; *SD*, standard deviation

#### Sphingolipids

##### Pathway and physiological role

Sphingolipids are essential components of cell membranes, and in neural tissue they are important in signal transduction and cell recognition. Fundamental building blocks of sphingolipids are long-chain bases (sphingoid bases). Their amine group is linked to an acyl group (e.g., fatty acid) and the backbone of the sphingoid base is linked with phosphate to an alcohol group (serine, ethanolamine or choline). The 3 principal forms of sphingolipids are ceramide and its derivatives sphingomyelin (SM) and glycosphingolipids [134].

##### Current research (Table 7)

Elevated plasma SM levels are an independent risk factor for CHD and an outcome predictor in patients with acute coronary syndrome [135, 136]. In lipid profiling of patients undergoing coronary angiography, 9 SM species are significantly associated with total and CHD long-term mortality (Table 7). Of 9 SM species, 5 showed an inverse association and 4 (specifically SM 16:0, 16:1, 24:1, and 24:2) displayed a positive association with total and cardiovascular mortality [132].

#### Acylcarnitines

##### Pathway and physiological role

Carnitine (3-Hydroxy-4-*N*-trimethylammoniobutanoate) is biosynthesized in the liver and kidneys from the amino acids lysine and methionine. Nutrition is an important, but not essential, source of carnitine. The highest concentrations of carnitine are found in red meat, but carnitine is also found in other foods such as nuts, seeds, legumes, vegetables, fruits and cereals. Fatty acid oxidation and thus fatty acid energy metabolism occur in the cellular mitochondria. In the cytosol, free fatty acids are attached with a thioester bond to coenzyme A (CoA). Carnitine transports long-chain acyl groups of fatty acids from the cellular cytosol through the mitochondrial membrane. On the outer mitochondrial membrane, carnitine acyltransferase l transfers acyl-CoA to the hydroxyl group of carnitine, resulting in acylcarnitine. The enzyme carnitine-acylcarnitine translocase catalyzes the transport of acylcarnitine to the inner mitochondrial membrane where carnitine acyltransferase II re-converts acylcarnitine to acyl-CoA [137].

##### Current research (Table8 )

Metabolic profiling of 165 polar metabolites was performed in hemodialysis patients. Individuals who died of a cardiovascular cause within 1 year of initiating hemodialysis revealed markedly higher concentrations of the 4 long-chain acylcarnitines oleoylcarnitine, linoleylcarnitine, palmitoylcarnitine, and stearoylcarnitine compared with survivors who had undergone hemodialysis for 1 year. Of these metabolites, oleoylcarnitine showed the strongest association with cardiovascular mortality after multivariable adjustment (Table 8) [138].

## Conclusions

Since many uncertainties remain in predicting all-cause mortality in patients hospitalized for CAP or for exacerbated COPD, additional prospective and retrospective observational studies are needed to ascertain other potential contributors to all-cause mortality in order to improve the prediction of initial high risk. Metabolomic analysis using MS may facilitate identification of outcome-specific metabolite signatures that could potentially generate new metabolic biomarkers of various organic systems as all-cause mortality predictors in CAP and in exacerbated COPD. Improvement in early and accurate identification of high-risk patients can help optimize short- and long-term management strategies in LRTI and as a result, potentially improve survival, leading to a more cost-effective allocation of medical resources. In addition, deeper knowledge of metabolic interactions in LRTI may provide new targets for future individual therapy and also be beneficial in predicting responses to therapy.

## Abbreviations

ACTH: Adrenocorticotropic hormone
ADMA: Asymmetric dimethylarginine
AKI: Acute kidney injury
Arg: L-arginine
AUC (or AUROC): Area under the receiver operating characteristic curve
BODE: Body mass index, airflow obstruction, dyspnea and exercise capacity index
CAP: Community-acquired pneumonia
CE: Capillary electrophoresis
CHD: Coronary heart disease
CI: Confidence interval
CKD: Chronic kidney disease
CoA: Coenzyme A
COPD: Chronic obstructive pulmonary disease
CRP: C-reactive protein
CURB-65: New-onset confusion, urea >7 mmol/L, respiratory rate ≥30 breaths/min, systolic or diastolic blood pressure <90 mmHg or ≤60 mmHg, respectively, age ≥65 years (pneumonia/LRTI risk scoring system)
CVD: Cardiovascular disease
DHEA: Dehydroepiandrosterone
DHEA-S: Dehydroepiandrosterone-sulfate
DHT: Dihydrotestosterone
eGFR: Estimated glomerular filtration rate
GC: Gas chromatography
GSB: Glutathione-S-bimane
GSH: Glutathione
FACS: Fluorescence-activated cell sorter
FC: Free cortisol
FT: Free testosterone
HD: Hemodialysis
HR: Hazard ratio
ICU: Intensive care unit
IDO: Indoleamine-2,3-dioxygenase
Kyn: Kynurenine
LC: Liquid chromatography
LRTI: Lower respiratory tract infection
lysoPC: Lysophosphatidylcholine
MS: Mass spectrometry
NO: Nitric oxide
NOS: Nitric oxide synthases
NT: Nitrotyrosine
NT-proBNP: Pro-B-type natriuretic peptide
OR: Odds ratio
PC: Phosphatidylcholine
PCT: Procalcitonin
PF: PaO2/FiO2
ProADM: Proadrenomedullin
PSI: Pneumonia severity index
RNS: Reactive nitrogen species
ROC: Receiver operating characteristics
ROS: Reactive oxygen species
SDMA: Symmetric dimethylarginine
SM: Sphingomyelin
TC: Total cortisol
TT: Total testosterone
TMA: Trimethylamine
TMAO: Trimethylamine-*N*-oxide
Trp: Tryptophan
Tyr: Tyrosine
